# Comparison and Efficacy of LigaSure and Rubber Band Ligature in Closing the Inflamed Cecal Stump in a Rat Model of Acute Appendicitis

**DOI:** 10.1155/2015/260312

**Published:** 2015-01-28

**Authors:** Chun-Chieh Yeh, Chia-Ing Jan, Horng-Ren Yang, Po-Han Huang, Long-Bin Jeng, Wen-Pang Su, Hui-Chen Chen

**Affiliations:** ^1^School of Medicine, China Medical University, Taichung 40402, Taiwan; ^2^Department of Surgery, China Medical University Hospital, Taichung 40402, Taiwan; ^3^Division of Transplantation, University of Illinois at Chicago, Chicago, IL 60612, USA; ^4^Department of Pathology, China Medical University Hospital, Taichung 40402, Taiwan; ^5^Department of Pathology, China Medical University Beigang Hospital, Yunlin 65152, Taiwan; ^6^Graduate Institute of Basic Medical Science, China Medical University, 91 Hsueh-Shih Road, Taichung 40402, Taiwan; ^7^Division of Hepatogastroenterology, Department of Internal Medicine, China Medical University Hospital, Taichung 40402, Taiwan

## Abstract

Safety of either LigaSure or rubber band in closing inflamed appendiceal stump in acute appendicitis has been less investigated. In this study, cecal ligation followed by resecting inflamed cecum was performed to mimic appendectomy in a rat model of acute appendicitis. Rats were sacrificed immediately (Group A) and 7 days (Group B) after cecal resection, respectively. The cecal stumps were closed by silk ligature (S), 5 mm LigaSure (L), or rubber band (R). Seven days after cecal resection, the LigaSure (BL) and silk subgroups (BS) had significantly less intra-abdominal adhesion and better laparotomy wound healing than rubber band subgroup (BR). The initial bursting pressure at cecal stump was comparable among the three methods; along with tissue healing process, both BL and BS provided a higher bursting pressure than BR 7 days after appendectomy. BL subgroup had more abundant hydroxyproline deposition than BS and BR subgroup. Furthermore, serum TNF-*α* in BR group kept persistently increasing along with time after cecal resection. Thus, the finding that LigaSure but not rubber band is safe in sealing off the inflamed cecal stump in rat model of acute appendicitis suggests the possibility of applying LigaSure for appendectomy via single port procedure or natural orifice transluminal endoscopic surgery (NOTES).

## 1. Introduction

Although single port laparoscopic appendectomy or natural orifice transluminal endoscopic surgery (NOTES) has been applied for the treatment of acute appendicitis [1, 2], technical difficulty limited their acceptance in clinical practice. For appendectomy, ligation and transection of mesoappendix and appendix are both needed. Though ligature, Endo-GIA, or clips could be currently conventional modalities used in ligation and transection of mesoappendix, their utilities for single port procedure or NOTES are limited due to narrow working space or high expenditure. Our preliminary experience suggested that either rubber band ligature or LigaSure were feasible ways to securely seal mesoappendix and appendiceal stump using the transumbilical single port or NOTES approach.

LigaSure had been examined for its safety in sealing off mesoappendix [3] or appendiceal stump in appendicitis [4]; however, its systemic influences and safety in sealing off appendiceal stump in acute appendicitis had not been investigated. In addition, by combining the flexible visual field and multiple working channel, gastroscopy has been applied in the NOTES [5]. Though rubber band ligature via gastroscopy has been widely applied in the ligation of esophageal or gastric varices [6], there is no study to investigate its safety in sealing off appendiceal stump.

Therefore, the objective of this study is to investigate the safety and effectiveness of using either LigaSure or rubber band ligature in closing appendiceal stump and mesoappendix for the treatment of acute appendicitis, whose results will then justify further investigations in applying these tools for appendectomy via single port procedure or NOTES.

## 2. Materials and Methods

### 2.1. Animals

**Figure 1:**
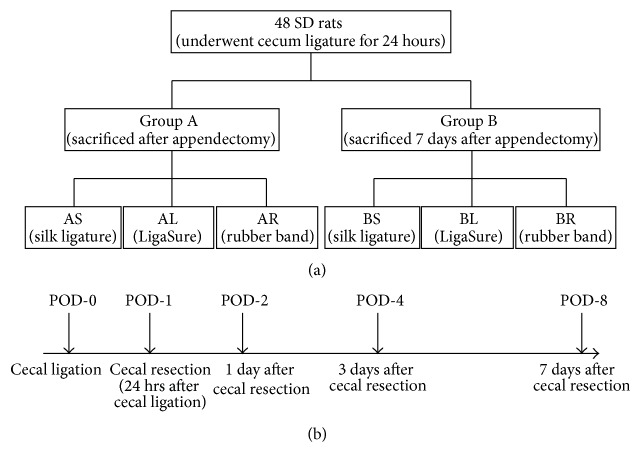
Scheme of experimental designs. (a) Grouping. (b) Flowchart of perioperative management. POD-0: postoperative day 0 when the cecal ligation was performed; POD-1: postoperative day 1 when the cecal resection was done 24 hours after cecal ligation; POD-2: postoperative day 2, 1 day after cecal resection; POD-4: postoperative day 4, 3 days after cecal resection; POD-8: postoperative day 8, 7 days after cecal resection.

Male Sprague-Dawley rats (weights ranged around 320 g) were purchased from the National Animal Center. All of rats were maintained in an animal facility at China Medical University. All rats underwent cecal ligation to mimic appendicitis in rat model on postoperative day 0 and received cecal resection to remove the obstructed cecal stump 24 hours after cecal ligation on postoperative day 1. Rats were randomly allocated into Group A and Group B. In Group A, we sacrificed the rats immediately after cecal resection. In Group B, instead of sacrificing the rats immediately, we kept the rats alive for 7 days till postoperative day 8. The scheme of grouping was shown in Figure 1(a); the flowchart of perioperative management was shown in Figure 1(b). All of the animal experiments were performed in accordance with the Guideline of the Institutional Animal Care and Use Committee of the China Medical University.

### 2.2. Experimental Model of Acute Appendicitis

Intraperitoneal ketamine hydrochloride (50 mg/kg body weight) combining with ether inhalation was used for anesthesia. All operations were performed under aseptic condition. After shaving for abdominal skin and sterilization for abdominal wall, a midline longitudinal laparotomy was performed. Cecum, which was measured around 1 cm in diameter and 2 cm in length, was identified. To create and mimic acute appendicitis in the rat model, ligation at the point proximal to the tip of cecum about 5 mm with 3-o silk was performed. By occluding the lumen of cecum, all rats suffered from cecal obstruction and inflammation that mimic acute appendicitis in clinical situation [4]. A single well-trained and experienced general surgeon was responsible for all of the following operations in the rats.

### 2.3. Appendectomy

In the “S” subgroup, the cecal stump and mesocecum were ligated together with 3-0 silk at both ends and transection line was put just in the middle of the two ties. In the “L” subgroup, 5 mm LigaSure precise (Valleylab, Boulder, CO, USA) device was used to seal off and transect the cecal stump and mesocecum. The cecal stump was transected till the formation of two adjacent white fusion lines at the proximal margin of cecal stump to seal off cecal lumen. Subsequently, the transection line was put distal to the two white lines and the distal lumen was also closed by coagulation line created by LigaSure. In the “R” subgroup, with the assistance of band ligator, two rubber bands (Frago impex sdn bhd, BENZ, Malaysia) were applied to the proximal end of the cecal stump and mesocecum, and then partial cecal resection was performed distally. Before application of rubber band, they were all sterilized by chlorhexidine as well as 75% alcohol and cleaned again with saline irrigation. The remnant distal length of cecal stump in all groups was around 1 mm in prevention of sloughing of rubber band or silk ligature. The cecal stumps were not buried into the remaining cecum, mimicking similar situation in laparoscopic appendectomy.

### 2.4. Evaluation of Wound Healing and Intra-Abdominal Adhesion

Laparotomy wound healing status and changes of body weight in the perioperative period were recorded serially. In addition, at the latest laparotomy, signs of bowel leakage, integrity of stump, presence of local abscess as well as peritonitis, and intra-abdominal adhesion condition were examined. The wound healing process was graded as follows: 1 = good healing without wound disruption, 2 = slightly poor healing with little gap at skin wound edge, and 3 = poor healing with obvious gap at wound edge and exposed subcutaneous layer. The grading of intra-abdominal adhesion was defined in accordance with a scale proposed by van der Ham et al. [7]. The scale was modified as follows: 1 = no adhesions, 2 = mild adhesion with momentum adhering to the cecal stump, 3 = moderate adhesion with small bowel adhering to cecal stump, and 4 = extensive and severe adhesion with surrounding bowel loops or abdominal wall adhering to the cecal stump, including local abscess formation. The pictures of intra-abdominal findings were photographed for evaluating wound healing status and intra-abdominal lesions by two independent well-experienced general surgeons.

### 2.5. Measurement of Bursting Pressure

In Group A after sacrificing the rats, a 2 cm ileocolic bowel segment containing cecum in the middle portion was taken down. The method of measurement of bursting pressure was modified from the protocol proposed by Elemen et al. [4]. Briefly, the bowel segment was ligated at colic end and one 18-gauge catheter was inserted into the other opening of the excised bowel segment. The catheter was connected to a 3-way stopcock, which was connected to a blood pressure monitor for simultaneously monitoring bursting pressure and to an infusion pump for injecting air into the bowel segment at a rate of 1 cc/min. The bursting pressure was recorded when the air bubble leaked from the closed bowel segment. To mimic true bowel loop situation, the content of bowel loop was not evacuated. In addition, to imitate intra-abdominal condition, a 2 cm ileocolic bowel segment along with adhered momentum, abdominal wall, and intestine was excised altogether for the measurement of summative bursting pressure.

### 2.6. Pathological and Immunohistochemical Examinations

After measuring the bursting pressure, the excised bowel segment was divided into two equally sized specimen containing part of the stump at each other. One of them was sent for histological examination after being stained by haematoxylin and eosin (H&E). The remnant bowel segment was stored in −80°C freezer and ready for the measurement of tissue hydroxyproline level. The wound healing condition at cecal stump was evaluated according to the modified Ehrlich/Hunt scale [8]; degree of inflammatory leukocytes infiltration, neovascularization, fibroblast aggregation, and collagen deposition were graded from 0 to 4 as follows: 0 = no evidence, 1 = occasion appearing, 2 = light scattering, 3 = abundant evidence, and 4 = confluent spreading. Two independent pathologists who did not know categorization of the specimens evaluated and scored the histological findings of wound healing status in resected cecal stumps.

### 2.7. Measurement of Hydroxyproline Concentration

The measurement of hydroxyproline level was conducted according to the protocol that has been published previously [9]. Briefly, after hydrolysis with 6 N HCL, the samples were oxidized with hydrogen peroxide and reacted with p-dimethylaminobenzaldehyde, and the absorbance of the product was determined at 540 nm. The amount of hydroxyproline is determined by finding the point corresponding to its optical density on the standard curve. The amount of the tissue hydroxyproline was expressed as *μ*g/g in wet tissue.

### 2.8. Detection of Serum TNF-*α*

To determine systemic inflammatory response in rats, we examined the serum concentration of tumor necrosis factor (TNF-*α*) by ELISA (enzyme-linked immunosorbent assay) with rat TNF-*α* ELISA kit (eBioscience). The procedure was conducted according to the instructions of the manufacturer.

### 2.9. Statistical Analyses

For categorical variables, Chi-square tests or Fisher exact test was conducted to analyze difference between groups, including intra-abdominal adhesion grade, laparotomy wound healing grade, and histological findings of wound healing status in cecal stumps. Cohen's Kappa test was used to measure agreement between two pathologists' evaluations on wound healing status of resected cecal stumps. For continuous variables, ANOVA tests with LSD post hoc analysis were utilized for calculation. All analyses used SAS software version 9.1 (by SAS Institute Inc., Cary, NC, USA). The statistically significant level was set at 0.05 by a two-tailed test.

## 3. Results

### 3.1. Body Weight Change

**Figure 2:**
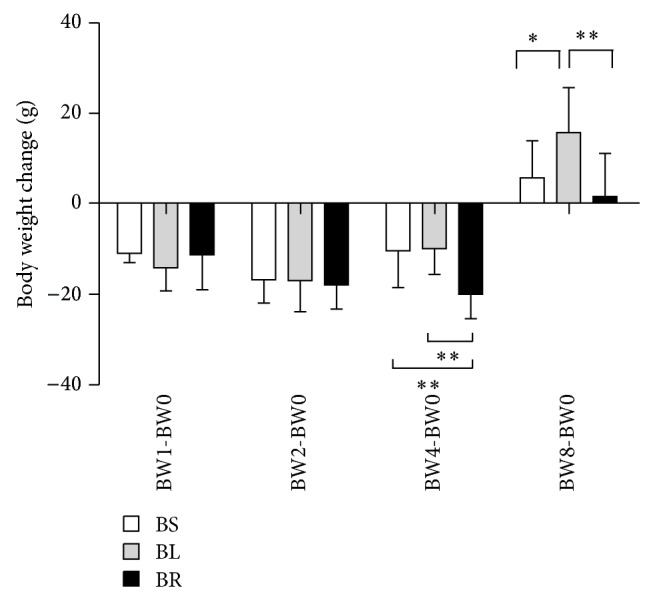
Changes of body weight in different subgroups. BW0: body weight measured on POD-0, BW1: body weight on POD-1, BW2: body weight on POD-2, BW4: body weight on POD-4, BW8: body weight on POD-8. The numbers represent the mean of body weight change ± SD from eight mice. ^*^ *P* < 0.05; ^**^ *P* < 0.01. Statistical significance was determined by ANOVA test and LSD post hoc analysis.

In Group A, body weight of rats on postoperative day 1 is less than that on postoperative day 0, and the differences between each subgroup were not statistically significant. In Group B, body weight of rats at each subgroup decreased after cecal ligation and recovered to more than baseline body weight on postoperative day 8. The magnitude of decrease in body weight at BR subgroup was the greatest among all the subgroups. On postoperative day 4, body weight of rats in BS and BL subgroups recovered better than that in BR subgroup (BW4–BW0; BS/BL/BR: −10.4 ± 8.1 g versus −9.9 ± 5.7 g versus −20.0 ± 5.4 g, *P* = 0.008) (Figure 2). On postoperative day 8, all rats in Group B recovered their body weight, and the increase of body weight in BL subgroup was significantly higher than that in both BS and BR subgroups (BW8–BW0; BS/BL/BR: 5.8 ± 8.2 versus 15.8 ± 10.0 g versus 1.6 ± 9.5 g, *P* = 0.018) (Figure 2). In other words, both LigaSure and silk ligature were superior to rubber band ligature in affecting body weight changes during recovering time after cecal resection.

### 3.2. Intra-Abdominal Findings

**Figure 3:**
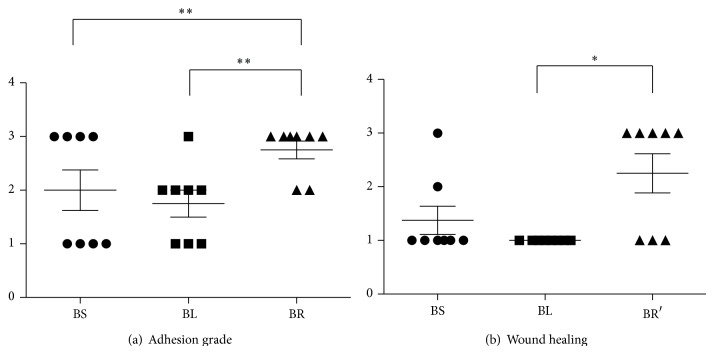
Intra-abdominal findings in different subgroups. (a) Intra-abdominal adhesion score. Intra-abdominal adhesion grade is as follows: grade 1 = no  adhesions, 2 = mild adhesion with momentum adhering to the cecal stump, 3 = moderate adhesion with small bowel adhering to cecal stump, and 4 = extensive and severe adhesion with surrounding bowel loops or abdominal wall adhering to the cecal stump, including local abscess formation. *N* = 8. ^**^ *P* < 0.01. Statistical significance was analyzed by Fisher exact test. (b) Laparotomy wound healing score. Laparotomy wound healing grade is as follows: grade 1 = good healing without wound disruption, 2 = slightly poor healing with little gap at skin wound edge, and 3 = poor healing with obvious gap at wound edge and exposed subcutaneous layer. *N* = 8. ^*^ *P* < 0.05; ^**^ *P* < 0.01. Statistical significance was analyzed by Fisher exact test.

The cecum in all rats after ligation was found to have omentum adhesion. Gangrenous change occurred at distal end of ligated cecum, which is compatible with clinical findings as acute appendicitis with gangrenous change. On postoperative day 8, 4 rats in BS subgroup and 6 rats in BR subgroup had severe intra-abdominal adhesion accompanied by local abscess near appendiceal stump; in contrast, only 1 rat in BL subgroup presented intra-abdominal local abscess. The proportion of severe intra-abdominal adhesion in BR subgroup is significantly higher than that in BS and BL subgroups (grade 4 adhesion in BS/BL/BR: 50% versus 12.5% versus 75%, *P* = 0.024) (Figure 3(a)). As for laparotomy wound healing status, 1 of 8 rats in BS subgroup and 5 of 8 rats in BR subgroup presented poor wound healing status with exposed subcutaneous muscular layer; however, all of 8 rats in BL subgroup had good wound healing status. The proportion of poor laparotomy wound healing in BR subgroup is significantly higher than that in BS subgroup (grade 3 laparotomy wound healing in BS/BL/BR: 12.5% versus 0 versus 62.5%, *P* = 0.024) (Figure 3(b)).

### 3.3. Bursting Pressure

In Group A, the bursting pressure of cecal stump was comparable between each subgroup (Table 1). In Group B, the bursting pressure of cecal stump of rats in BS subgroup was comparable to that in BL subgroup, but significantly higher than that in BR subgroup (Table 1). In other words, the initially mechanical force to close appendiceal stump was comparable among the three methods. By contrast, after tissue repairing, both LigaSure and silk ligature provided a better sealing effect than rubber bands.

In addition, bursting pressure of BL subgroup was higher than that of AL subgroup (136.1 ± 49.8 versus 87.0 ± 64.9 mmHg, *P* = 0.032), but bursting pressure between BS and AS subgroups (137.8 ± 24.5 versus 134.7 ± 34.9 mmHg, *P* = 0.883) as well as that between BR and AR subgroups (96.3 ± 15.9 versus 84.3 ± 33.6 mmHg, *P* = 0.570) was comparable. In other words, after tissue healing, LigaSure could provide stronger strength to seal cecal stump when compared to the initially mechanical sealing force.

### 3.4. Hydroxyproline Level

**Figure 4:**
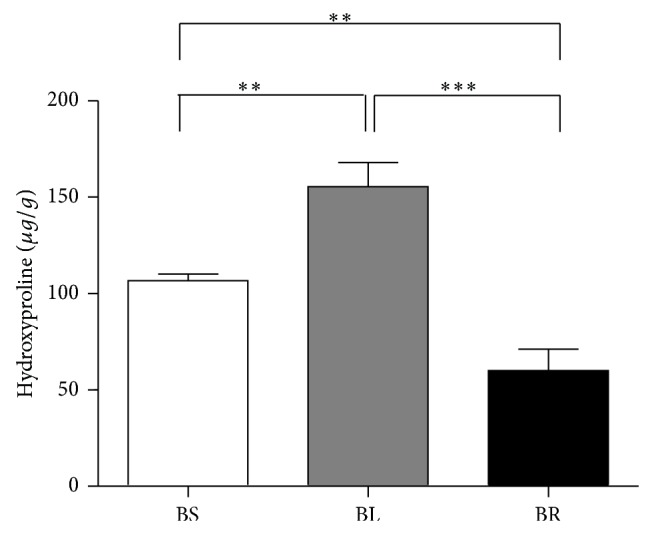
Content of tissue hydroxyproline in the cecal stump in different subgroups. The numbers represent the mean of content of hydroxyproline in the cecal stump (*μ*g/g of wet tissue) ± SD from eight mice. ^**^ *P* < 0.01; ^***^ *P* < 0.001. Statistical significance was determined by ANOVA test and LSD post hoc analysis.

Analysis of hydroxyproline level in Group B showed that the amount of collagen deposition near the cecal stump in BL subgroup was the highest, followed by BS and BR subgroup (BS/BL/BR: 106.8 ± 9.6 versus 155.6 ± 35.4 versus 60.1 ± 31.2 *μ*g/g wet tissue, *P* < 0.001) (Figure 4).

### 3.5. Systemic Proinflammatory Cytokines

**Figure 5:**
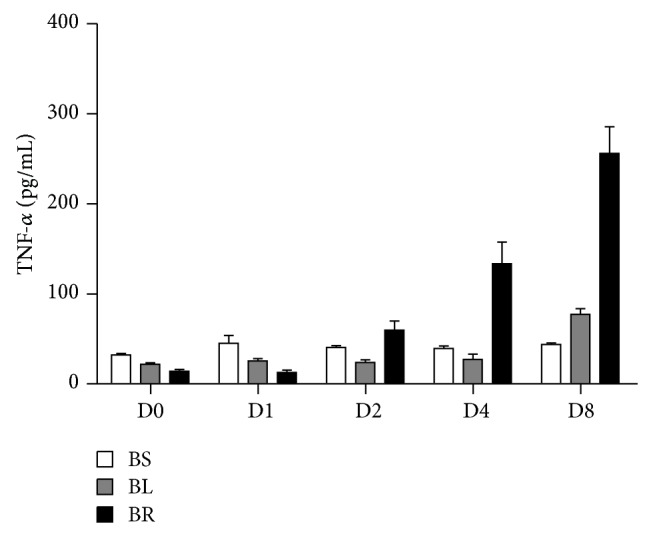
Levels of systemic proinflammatory cytokine, TNF-*α*, in different subgroups. D0: POD-0; D1: POD-1; D2: POD-2; D4: POD-4; D8: POD-8. The numbers represent the mean of concentration of TNF-*α* in the serum (pg/mL) ± SD from eight mice. Statistical significance was determined by ANOVA test and LSD post hoc analysis.

The systemic proinflammatory cytokine, such as serum TNF-*α*, was serially analyzed in Group B. The TNF-*α* levels in BR subgroup showed a significant increasing trend along with time and reached to the highest level on postoperative day 8 (postoperative day 0/1/2/4/8: 14.4 ± 6.5 versus 13.2 ± 7.0 versus 60.2 ± 28.0 versus 134.0 ± 67.7 versus 256.0 ± 84.1 pg/mL, *P* < 0.001). Additionally, the serum TNF-*α* of BR subgroup on postoperative day 8 was significantly higher than that of either BS or BL subgroup (BS/BL/BR: 44.0 ± 4.8 versus 77.4 ± 18.6 versus 256.0 ± 84.1 pg/mL, *P* < 0.001) (Figure 5).

### 3.6. Histological Examination for Appendiceal Stump Healing

**Figure 6:**
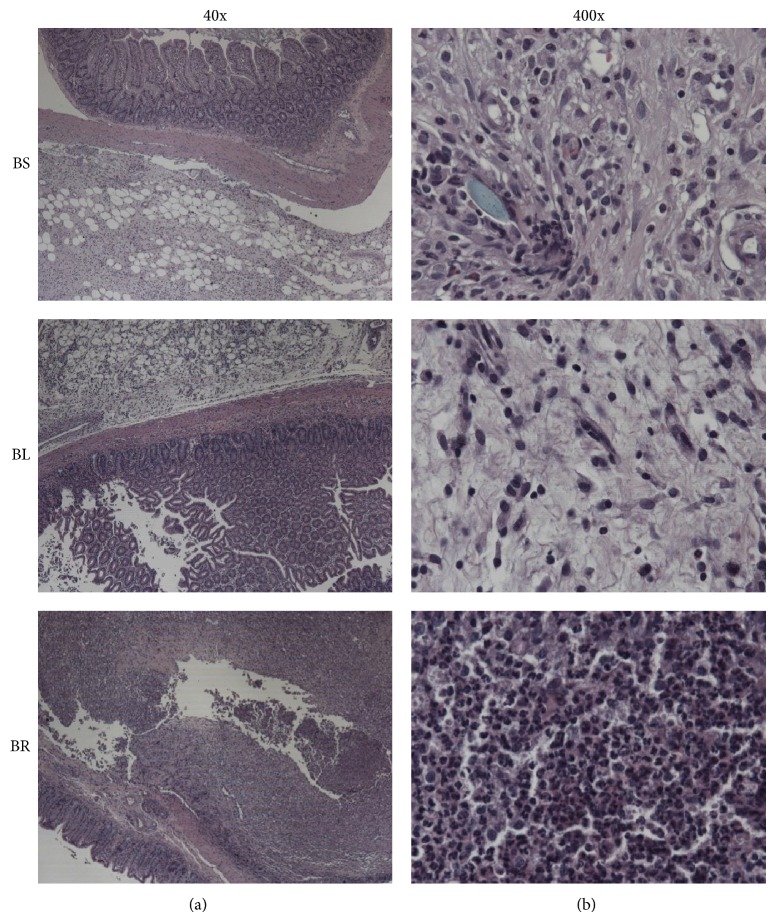
Examination of cell infiltration at cecal stump in different subgroups. Representative cecal stump sections stained with haematoxylin and eosin (H&E) from BS, BL, and BR. The figures were photographed at 40x (a) and 400x (b) magnification.

The microscopic views at cecal stump sections stained with haematoxylin and eosin (H&E) in BS, BL, and BR subgroups were shown in Figure 6. Using the modified Ehrlich-Hunt scale [8], the results of microscopic exams were shown as follows: in Group B (BS, BL, and BR), the degree of leukocyte infiltration in BR group was significantly higher than that in BS and BL groups (proportion of leukocyte infiltration score 4 in BS/BL/BR, 25% versus 25% versus 75%, *P* = 0.041), whereas the differences in fibroblast activity, collagen deposition, and neovascularization scores within Group B were not significant. In addition, there was no significant difference in the four scores within Group A (AS, AL, and AR). The kappa score was 0.7.

## 4. Discussion

Single port laparoscopic appendectomy for the treatment of acute appendicitis has been developed but not popular, because most of them need specially designed curve instrument [10, 11]. LigaSure and rubber band ligature have not been investigated as a tool to ligate mesoappendix and appendiceal stump for the treatment of acute appendicitis. Herein, we used a rat model of appendicitis to investigate safety and feasibility of appendectomy in inflamed and gangrenous appendix with either LigaSure or rubber band ligature. The results indicated that LigaSure is as effective and safe as conventional silk ligature in sealing inflamed cecal stump. By contrast, though rubber band ligature could provide sufficient mechanical sealing force at early stage after cecal resection, it would also cause poor healing status in the cecal stump due to exaggerated local inflammation and abscess formation as well as less collagen deposition. Furthermore, rubber band ligature itself also led to severer systemic inflammation and poor recovery in body weight.

Wound healing involves a series of reactions, including initial inflammation after injury or infection, followed by recruitment of inflammatory cells, such as neutrophil and macrophage, fibroblast, and collagen deposition [12]. The amount of collagen deposition is measured by hydroxyproline content in the surrounding area of healing wound and more collagen deposition means better wound healing process [12]. The BL subgroup had the most abundant content of hydroxyproline in comparison with BS and BR subgroups; that means that wound healing process in the BL subgroup is the best among the three methods. In addition, the bursting pressure of cecal stump is the most direct measurement of mechanical strength in the sealed stump. In Group A, bursting pressure of cecal stump was comparable between AS, AL, and AR subgroups. In other words, the direct mechanical force to seal off appendix was comparable between the three tools. However, after 7-day healing process, the bursting pressure of cecal stump in the rats at BS and BL subgroups was significantly higher than that at BR subgroup. Therefore, taking healing process into consideration, the bursting pressure of BR subgroup was lowest, even though the bursting pressure of BR subgroup was still higher than 25 mmHg, which was normal intraluminal pressure of small bowel loop [13].

Ligature in cecum of a rat has been accepted as a rat model of acute appendicitis [4]. Additionally, the result indicated that serum concentration of TNF-*α* measured before and 1 day after cecal ligation did not differ significantly which implied early appendicitis could mainly be a locally inflammatory illness, and the finding was also consistent with that proposed in previous human study [14]. Furthermore, the study indicated an increasing trend of serum TNF-*α* in BR subgroup, and we believed that rubber band related foreign body reaction might cause persistently increased TNF-*α* in BR subgroup. More inflammatory cells infiltrating in cecal stump observed at BR subgroup might also support this hypothesis. In addition, rubber band ligation related tissue ischemia could also contribute to release of HMGB1, a DAMP (damage associated molecular pattern), and subsequently cause prolonged TNF-*α* production [15]. The persistent inflammatory status, inferred by increased TNF-*α*, might also be responsible for delayed resumption of oral intake and recovery of body weight in the rats at BR subgroup [16].

Elemen et al. had conducted an excellent experimental study to investigate safety of LigaSure in sealing off appendiceal stump following appendectomy for appendicitis [4]. Because the study lacked observing systemic influences, such as body weight changes as well as serum TNF-*α* and quantification of local collagen deposition around appendiceal stump among various treatment modalities, we conducted this study to further address this issue. According to clinical observation, overwhelming release of proinflammatory cytokines could cause profound septic shock in patients with appendicitis. Therefore, our study provided important and clinically relevant information regarding the systemic influences and safety of LigaSure and rubber band ligature in closing appendiceal stump in rat model of acute appendicitis.

Because of lack of well-designed and safe sealing tools in bowel walls, single port appendectomy or NOTES in abdominal surgery was not prevailing yet in current surgical field. After proving the safety of LigaSure in transecting and sealing off mesoappendix [3] and cecal stump in this study, LigaSure should be further applied in large animal study or human clinical trials to investigate its feasibility and safety in either NOTES or single port operation for the treatment of acute appendicitis.

## 5. Conclusion

In this study, we identified that LigaSure may be a safe and convenient tool in transecting mesoappendix and appendiceal stump in rat appendicitis model. Rubber band ligature should not be suitable to close appendiceal stump due to profound systemic and local inflammation as well as associated impaired wound healing process at the appendiceal stump.

## Figures and Tables

**Table 1:** Bursting pressure in different experimental groups.

	AS	AL	AR	*P* value^a^	Post hoc
Bursting pressure (mean ± SD, mmHg)	134.7 ± 34.9	87.0 ± 64.9	84.3 ± 33.6	0.137	

	BS	BL	BR	*P* value^a^	Post hoc

Bursting pressure (mean ± SD, mmHg)	137.8 ± 24.5	136.1 ± 49.8	96.3 ± 15.9	**0.034**	BS = BL > BR

^a^Statistical analysis was conducted using ANOVA and Bonferroni post hoc analysis. Significant differences are represented in boldface.
